# A Single Dose of Synbiotics and Vitamins at Birth Affects Piglet Microbiota before Weaning and Modifies Post-Weaning Performance

**DOI:** 10.3390/ani11010084

**Published:** 2021-01-05

**Authors:** Marion Girard, Marco Tretola, Giuseppe Bee

**Affiliations:** Agroscope, Tioleyre 4, 1725 Posieux, Switzerland; marco.tretola@agroscope.admin.ch (M.T.); giuseppe.bee@agroscope.admin.ch (G.B.)

**Keywords:** piglet microbiota, probiotics, vitamins, prebiotic, post-weaning performance

## Abstract

**Simple Summary:**

For pig producers, enhancing piglet performance and reinforcing their health is crucial to ensure the optimal development and welfare of the animals, and to reduce the use of antimicrobials. This study investigated the effect of a single-dose application of a supplement on piglet growth and health, and on their microbiota in the suckling period and after weaning. At birth, piglets from eight litters received a supplement containing two probiotic strains, prebiotics, vitamins, and immunoglobulins, while piglets from six other litters received a dose of water. The supplement given at birth improved post-weaning piglet growth and reduced post-weaning diarrhea. These better post-weaning performances seem to be related to slight changes in the microbiota in the suckling period but not in the post-weaning period. In the suckling period, supplemented piglets shared some growth-related taxa, such as bacteria from the *Lactobacillus* genus, that unsupplemented piglets did not share. The present study highlights the importance of early-life microbial colonization on the subsequent performance and health of piglets.

**Abstract:**

Early-life microbial colonization is an important driver for the development and maturation of the gut. The present study aimed to determine whether a single-dose supplement given only at birth would improve piglet performance and modify their fecal microbiota during the suckling and post-weaning periods. At birth, piglets from eight litters received a supplement (SUP+) while piglets from six other litters received water (SUP−). All piglets were monitored until two weeks post-weaning, and fecal samples were collected on Day 16 of age and two weeks post-weaning (Day 39 ± 1). The supplementation resulted in an improvement of average daily gain during the whole experimental period, mainly due to a better growth and a reduction in the incidence of diarrhea in the post-weaning period. There were no differences in the abundance and diversity of the main taxa, although the supplementation increased the relative abundance of rare taxa, such as bacteria from the Saccharibacteria and Cyanobacteria phyla, and the Lentisphaeria class in the suckling period. In addition, at 16 days of age, SUP+ piglets had a more diverse core microbiota, with bacteria from the *Lactobacillus* genus being present in the core microbiota of SUP+ piglets and absent from SUP− piglets. Therefore, the enhanced growth performance and reduction in diarrhea seem to be related to changes in fecal microbiota during the suckling period rather than at two weeks post-weaning.

## 1. Introduction

In recent decades, advances in genetics have increased the prolificacy of sows, resulting in an increase in litter size [1,2]. Nowadays, giving birth to more than 16 piglets is common with hyperprolific sows [3]. Nevertheless, this improvement has concomitantly decreased the average litter birth weight and increased the weight variability between and within litters, as well as the proportion of low-birth-weight piglets [4]. As a consequence, the average daily gain (ADG) of piglets born from large litters has also decreased [5]. In addition, small piglets are more sensitive to cold due to less body energy reserves and are less combative to access the best teats [6]. Therefore, their immune and nutritional status are affected, which in turn compromises their growth performance and increases the risk of morbidity and mortality. Improving the survival rate, growth, and resistance to diseases of all piglets, in particular low birth weight piglets, is essential to improve piglet health and sow productivity. It is well recognized that early-life gut colonization plays a central role in the development and the maturation of the gut, thereby improving the development and disease resistance of piglets [7]. Besides a sufficient colostrum and milk intake, several nutritional substances such as probiotics, prebiotics, and vitamins have proven to be effective in enhancing gut colonization and gut health. The use of synbiotics is gaining interest since it combines probiotics, which are live microorganisms, and prebiotics, which are non-digestible dietary fibers [8]. This combination improves survival rate and favors the growth and activity of beneficial microorganisms in the gut. 

The fermentation of dietary fibers by microorganisms in the gut produces volatile fatty acids (VFAs), mainly acetate, propionate, and butyrate, that animals utilize as an energy source. Specifically, VFAs produced in the large intestine contribute up to 11% of the total energy requirements in pigs, while the VFAs produced in the total hindgut contribute up to 25% of the energy requirements [9]. In particular, some VFAs like butyrate, are involved in the development of colonocytes, which leads to proper gut maturation [10,11]. They have also been shown to reduce the growth of potential pathogens (e.g., *Clostridium* and *Salmonella*) and to promote the growth of beneficial bacteria (e.g., *Lactobacilli* spp.) by decreasing the pH of the gut content [12]. In addition, some vitamins and trace elements have been shown to stimulate immune functions [11,12,13,14]. For instance, vitamin A and its metabolite, retinoic acid, are involved in the development of gastrointestinal immune responses to antigens, whereas vitamin E and selenium have a synergistic effect on immunoglobulin production in addition to antioxidant properties [13,14,15,16]. Thus, the modulation of gut microbiota by the use of synbiotics and vitamins early in life could represent a promising strategy to improve the energy supply of the piglets and to avoid or reduce the detrimental effects of post-weaning syndrome.

This study intended to determine the effect of a supplementation consisting of synbiotics, vitamins A and E, selenium, and immunoglobulins given at birth on the development of piglets from birth to two weeks post-weaning, and the effects on their fecal microbiota.

## 2. Materials and Methods 

### 2.1. Animals, Housing and Experimental Design

The animal experiment and all interventions on piglets were approved by the Swiss cantonal veterinary office (approval number: 2018_15E_FR) and were in compliance with Swiss guidelines for animal welfare. On Day 106 (±2) of gestation, 14 Swiss Large White sows from two farrowing series were individually housed in loose farrowing pens of 7.1 m^2^, consisting of 6.0 m^2^ of a concrete floor covered with wheat straw and 1.1 m^2^ of a galvanized steel floor. In the farrowing pens, piglets had access to a covered, heated area (1.37 × 0.60 m^2^) from birth to two weeks post-weaning. All animals had access to clean water. Parturition was induced when gestation time exceeded 115 days for primiparous sows and 116 days for multiparous sows by injection of 1 mL (2 times 0.5 mL) of Estrumate^®^ (Provet AG, Lyssach, Switzerland). In the first 10 h after the end of farrowing and after colostrum suckling, piglets from eight litters were orally offered 2 mL of a supplement (SUP+; Sanobiotic^®^ duo-FG, Zehetmayer AG, Winden, Switzerland), whereas piglets from six other litters received 2 mL of water (SUP−). Treatment group allocation was performed prior to farrowing based on parity (3.7 ± 1.2; mean ± standard deviation [SD]) and on sow body weight on Day 110 of gestation (298 ± 24 kg; mean ± SD). Cross fostering of male piglets within the same treatment group was allowed in the first two days of life to standardize litters to 12 ± 1 (mean ± SD) piglets on average. 

### 2.2. Diets, Feeding and Composition of the Supplement

Diets were formulated according to the Swiss feed recommendations for pigs [17]. From farrowing to weaning, sows were fed a standard lactation diet containing 189 g of crude protein, 38 g of crude fiber, 11.4 g of lysine, and 14.1 MJ of digestible energy per kg of feed. The amount of feed was adapted to meet the sow requirements according to the number of suckling piglets. Along the experiment, piglets had no access to sow diet. The supplement, received by the SUP+ piglets, was a mixture of the two probiotics, *Enterococcus faecium* (E1705) and *Saccharomyces cerevisiae* (E1703), inulin, immunoglobulins, vitamins, and selenium. The analyzed chemical composition of the supplement was 835 g of dry matter, 150 g of crude protein, 351 g of fat, 4 g of crude fiber, 39,875 mg of vitamin E, 1,362,500 IU of vitamin A, 256,000 IU of vitamin D3, 48 mg of selenium, 5 × 10^12^ CFU of *S. cerevisiae* (E1703), and 3 × 10^12^ CFU of *E. faecium* (E1705) per kg of supplement. From Day 18 ± 1 onwards, piglets had access to a post-weaning diet containing 170 g of crude protein, 50 g of crude fiber, 11.5 g of lysine, and 14.0 MJ of digestible energy per kg of feed.

### 2.3. Measurements, Sampling and Volatile Fatty Acid Analysis

On Day 16 and two weeks post-weaning (Day 39 ± 1), feces were collected from two female piglets per litter to further determine microbiota and quantify the level of VFAs. The VFA profile in the feces was determined by high-performance liquid chromatography (HPLC) with a method adapted from Htoo et al. [18]. Briefly, feces samples that had been previously weighed and frozen at −20 °C with 1 mL of phosphoric acid (25%, *w*/*v*) were thawed. Following defrosting, 1 mL of internal standard (pivalic acid at 1%, *w*/*v*) and 18 mL of distilled water were added into the tube. This preparation was stirred for 3 h at room temperature before being centrifuged for 5 min at 4000 g. The supernatants were filtered and analyzed for VFA level using HPLC (Ultimate 3000, Thermo Fisher Scientific, Reinach, Switzerland) with an exchange ion column (Nucleogel ION 300 OA 300 × 7.8 mm, Marcherey-Nagel AG, Oensingen, Switzerland) and equipped with a refractive index detector (RefractoMax 521, Thermo Fisher Scientific, Reinach, Switzerland). During the experiment, growth performance of the selected female piglets was recorded by weighing them on Days 0 (at birth), 2, 5, 16, 25 ± 1 (weaning), 32 ± 1, and 39 ± 1 after birth. From weaning to Day 39 (±1), piglets were not mixed and stayed in the farrowing pens.

### 2.4. DNA Extraction and Microbiota Profiling

Feces previously stored at −80 °C were thawed, and total genomic DNA was extracted using a QIAamp Fast DNA Stool Mini Kit (QIAGEN GmbH, Hilden, Germany) according to the manufacturer’s instructions. Microbial profiling was determined by high-throughput sequencing of the V3-V4 region of the bacterial 16S rRNA gene. Two-step Nextera polymerase chain reaction (PCR) libraries using the primer pair 341F (5′-CCT ACG GGN GGC WGC AG-3′) and 802R (5′-GAC TAC HVG GGT ATC TAA TCC-3′) were created. Subsequently, the Illumina MiSeq platform and a v2 500 cycle kit (San Diego, CA, USA) were used to sequence the PCR libraries. The produced paired-end reads, which passed Illumina’s chastity filter were subject to de-multiplexing and trimming of Illumina adaptor residuals using Illumina’s real-time analysis software included in the MiSeq reporter software v2.6 (no further refinement or selection). The quality of the reads was checked with the FastQC software v0.11.8. The locus-specific V34 primers were trimmed from the sequencing reads with the Cutadapt software v2.3. Paired-end reads were discarded if the primer could not be trimmed. Trimmed forward and reverse reads of each paired-end read were merged to in-silico reform the sequenced molecule considering a minimum overlap of 15 bases using the USEARCH software v11.0.667. Merged sequences were then quality filtered, allowing a maximum of one expected error per merged read. Reads that contained ambiguous bases or were outliers regarding the amplicon size distribution were also discarded. The remaining reads were denoised using the UNOISE algorithm implemented in USEARCH to form operational taxonomic units (OTUs), which discarded singletons and chimeras in the process. The resulting OTU abundance table was then filtered for possible bleed-in contaminations using the UNCROSS algorithm, and abundances were adjusted for 16S copy numbers using the UNBIAS algorithm. OTUs were compared against the reference sequences of the RDP (Ribosomal Database Project) 16S database, and taxonomies were predicted considering a minimum confidence threshold of 0.5 using the SINTAX algorithm implemented in USEARCH.

### 2.5. Statistical Analyses

Weights, ADG, VFA production, and days of diarrhea were analyzed with the MIXED procedure of the SAS software v9.4 (SAS Institute Inc., Cary, NC, USA), and occurrence of diarrhea was analyzed with the GLIMMIX procedure of SAS. Due to a non-normal distribution of the residuals, weight data were converted in logarithm prior to analysis. All models considered piglets as experimental units and included the effect of the supplementation, farrowing series, day (when appropriate), and interaction of the supplementation × day (when appropriate) as fixed effects, and the sow as a random effect. For data measured over days on the same piglet, a repeated measurement statement was included in the models, assuming a first-order autoregressive covariance structure for evenly spaced days (i.e., for VFA profile and occurrence of diarrhea) or a spatial power covariance structure for non-evenly spaced days (i.e., for weights). The SLICE option of the MIXED procedure in the SAS software was used to assess the supplementation effect within each day. Differences were considered significant if *p* ≤ 0.05 and considered a tendency if 0.05 < *p* ≤ 0.10. For a significant effect, the PDIFF (*p*-values for differences of the LS-means) option with an adjustment for the Tukey–Kramer test was used to separate least squares means. In the tables and figures, data are reported as least squares means and pooled standard error of the mean (SEM). For gut microbiota data, alpha diversity was estimated using the Richness (Observed and Chao1), Simpson and Shannon indices. Beta diversity was calculated using the weighted and unweighted Unifrac distance methods on the basis of rarefied OTU abundance counts per sample. Additionally, the variance (PERMANOVA) and similarities (ANOSIM) of the tested groups were analyzed. Alpha and beta diversity calculations and the rarefaction analysis were performed with the R software packages phyloseq v1.26.1 and vegan v2.5–5. The linear discriminant analysis effect size (LEfSe) was performed to determine statistical differences in taxa abundance between groups by using the following conditions: the alpha value for the non-parametric factorial Kruskal–Wallis sum-rank test among the classes was < 0.05 and the threshold on the logarithmic linear discriminant analysis score for the discriminative features was > 3.0 [19]. The “microbiome” library was used to estimate the common core microbiota, with a detection threshold of 0.001 and prevalence in 80/100 samples. All data analyses for microbiota evaluation were performed in R v2.5.0 (Boston, MA, USA). Multivariate analysis by linear models was conducted using MaAsLin [20] to test for associations of microbial abundances (at all taxonomic levels from domain to genus) with fecal VFA content. Default settings were used for this analysis. Specifically, in the analysis, only taxa with prevalence > 0.01 across samples and with a significance threshold of 0.05 were included.

## 3. Results

### 3.1. Growth Performance and Diarrhea

**Figure 1:**
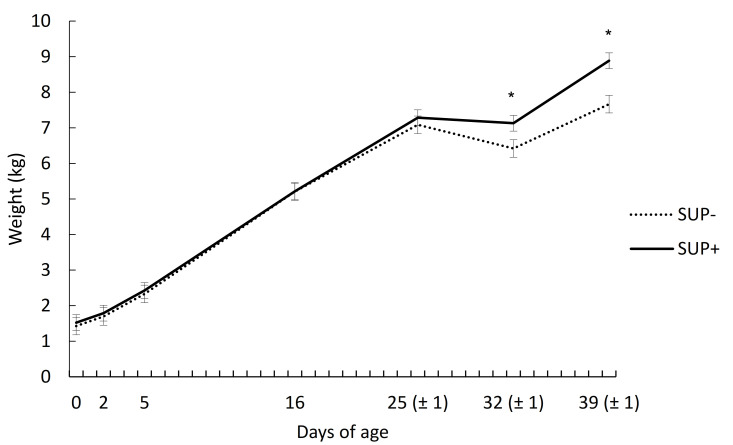
Development of body weight from birth to Day 39 of age of piglets from the unsupplemented (SUP−) or supplemented (SUP+) group. The supplement, given at birth, consisted of a mixture of probiotics, prebiotics, immunoglobulins, vitamins, and selenium. The supplementation × day interaction was *p* = 0.05. * indicates a difference (*p* < 0.05) within a day.

The weights between the two groups were similar each day from birth to weaning, but after weaning, SUP+ piglets were heavier (*p* < 0.05) than SUP− piglets, resulting in a difference of 1.2 kg at two weeks post-weaning (Figure 1; significant supplementation × days interaction).

The ADG from birth to weaning did not differ (*p* = 0.98) between the two treatment groups. However, from birth to Day 39 of age, SUP+ piglets had a 26 g/d greater (*p* = 0.05) ADG than SUP− piglets. The better performance of SUP+ piglets can be explained by the lower (*p* = 0.04) body weight loss in the first week post-weaning (Table 1). 

In both groups, piglets had diarrhea for 3.7 days on average (*p* = 0.22). However, the occurrence of diarrhea was or tended to be lower (*p* ≤ 0.10) in SUP+ piglets than in SUP− piglets in the first and second post-weaning week. Thus, SUP+ piglets had 6.8% units lower (*p* = 0.02) diarrhea incidence in the pre- and post-weaning period compared with SUP− piglets.

### 3.2. Production of Volatile Fatty Acids

The supplementation had no effect (*p* > 0.10) on the total VFA content or the proportion of each VFA. However, the day had a significant effect (*p* < 0.01) on the aforementioned parameters. After weaning, the production of VFAs increased, as well as the proportions of acetate and propionate. On the contrary, the proportions of butyrate, valerate, and the two branched-chain fatty acids, isobutyrate and iso-valerate, decreased from the pre-weaning to the post-weaning period (Table 2).

### 3.3. Microbial Diversity and Microbiota Composition in the Feces

A total of 1064 OTUs obtained by 9,325,894 sequences were acquired by the high throughput sequencing. The supplementation did not affect (*p* > 0.10) the indices of α-diversity given by the richness (observed Species or Chao1 index), the evenness (Shannon or Simpson indexes), or the phylogenetic diversity (Supplementary Material Figure S1). Similarly, both the weighted and unweighted β-diversity were similar (*p* > 0.10) between SUP+ and SUP− piglets (Supplementary Material Figure S2). Regardless of the supplementation, piglets at two weeks post-weaning had greater (*p* < 0.001) indices of α-diversity and phylogenetic diversity than on Day 16 of age (Supplementary Material Figure S1). In addition, both weighted and unweighted β-diversity results show that samples clustered (*p* < 0.05) according to age, and were therefore different between Day 16 of age and two weeks post-weaning, with PC1 explaining 46.7% of the variation and PC2 explaining 14.8% in the Weighted UniFrac analysis (Supplementary Material Figure S2A,B).

**Figure 2:**
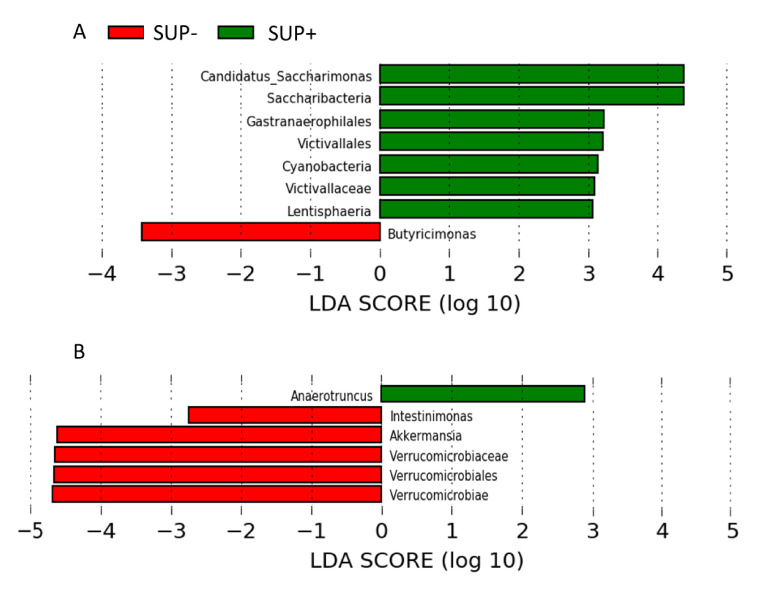
Linear discriminant analysis (LDA) coupled with effect size measurements (LEfSe): The most differentially abundant taxa found in stool samples of unsupplemented piglets (SUP−; in red, N = 13) or piglets supplemented (SUP+; in green, N = 16) at birth with a mixture of probiotics, prebiotics, immunoglobulins, vitamins, and selenium at (**A**) 16 days of age or (**B**) two weeks post-weaning.

A total of 19 phyla, 30 classes, 34 orders, 50 families, and 125 genera of bacteria were identified during the whole experiment. Several differences in the relative abundance of different taxa occurred on 16 days of age between SUP+ and SUP− piglets. Bacteria from the *Candidatus Saccharimonas* genus, belonging to the Saccharibacteria phylum, together with the Gastranaerophilales order, belonging to the Cyanobacteria phylum were more abundant (*p* = 0.02 and *p* = 0.01, respectively) in the feces of SUP+ piglets compared with SUP− piglets (6.33 and 2.37 log2 fold change (FC), respectively). Similarly, bacteria from the *Victivallaceae* family, belonging to the Victivallales order and Lentisphaera class, were more represented (*p* = 0.004) in SUP+ piglets than in SUP− piglets (4.23 log2 FC). On the contrary, bacteria from the *Butyricimonas* genus were less abundant (*p* = 0.03) in SUP+ piglets compared with SUP− piglets (−2.24 log2 FC; Figure 2A, Table 3). 

Two weeks post-weaning, the abundance of some bacteria at the genus level differed between the two treatment groups. Specifically, bacteria from the *Intestinimonas* and *Akkermansia* genera, the latter belonging to the Verrucomicrobiae class, were under-represented (*p* = 0.02, −3.14, and −9.48 log2 FC, respectively) in SUP+ piglets. Interestingly, bacteria from the *Anaerotruncus* genus were found in the SUP+ group, while it was absent in all samples belonging to the SUP− group (Figure 2B, Table 3).

**Figure 3:**
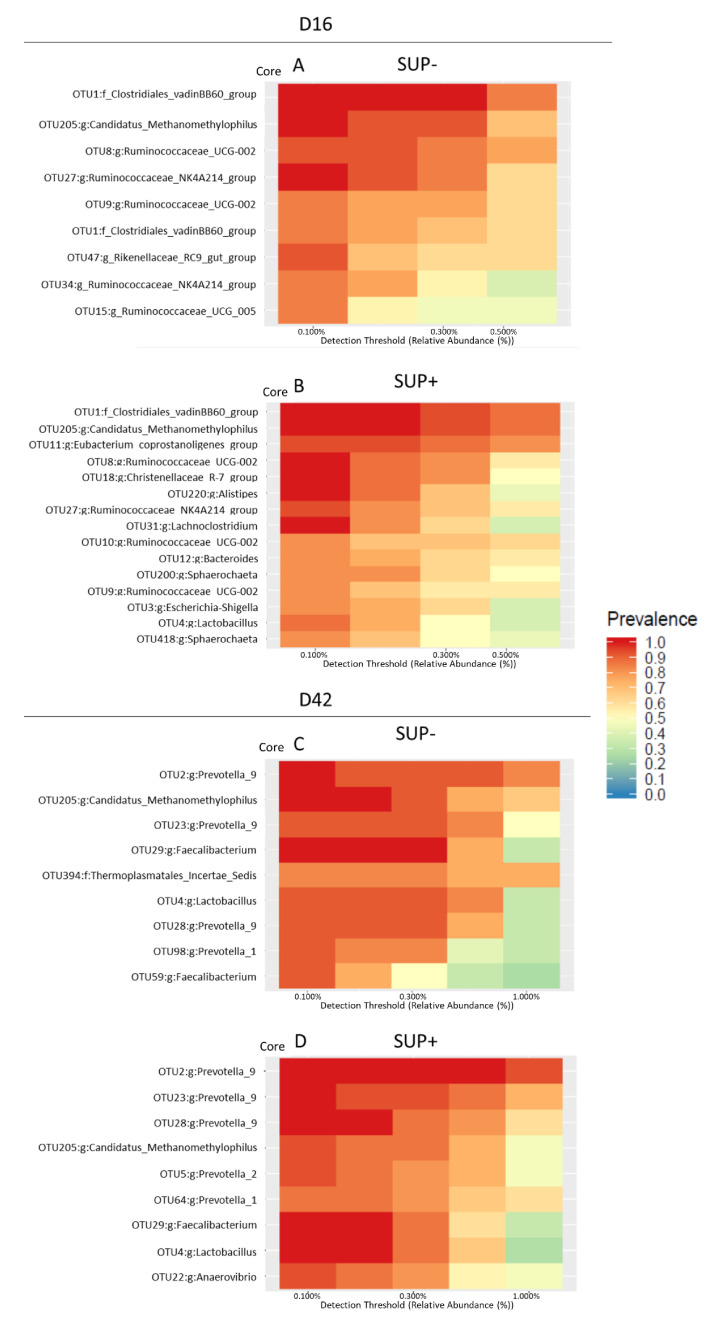
Core microbiota heatmaps showing bacteria shared by 80% of unsupplemented piglets (SUP−) or piglets supplemented (SUP+) at birth with a mixture of probiotics, prebiotics, immunoglobulins, vitamins, and selenium at Day 16 of age (**A**,**B**) and two weeks post-weaning (**C**,**D**).

Regardless of the supplementation, as expected, the microbial communities varied between 16 days of age and two weeks post-weaning. The age-related differences in the microbial population are reported in Supplementary Figure S3. The effect of supplementation on the size of the core microbiota was evaluated comparing the SUP− and SUP+ groups on Day 16 of age and two weeks post-weaning. On Day 16 of age, a total of nine (0.84%) and 15 (1.4%) OTUs were shared among 80% of SUP− and SUP+ piglets, respectively (Figure 3A,B). Specifically, the SUP+ group core microbiota contained members from several genera, including *Eubacterium coprostanoligenes*, *Christensenellaceae R-7 group*, *Alistipes*, *Lachnoclostridium*, *Ruminococcaceae_UCG*, *Bacteroides*, *Spaerochaeta*, *Escherichia-Shigella*, *Lactobacillus*, and *Sphaerochaeta*, which were not part of the core microbiota of the SUP− group. On Day 39, the two groups showed the same core microbiota size (nine OTUs), but some differences were present (Figure 3C,D). For example, the core microbiota of SUP− piglets was characterized by the presence of the *Thermoplasmatales incertae sedis* family, while the *Anaerovibrio* and *Prevotella 2* genera were found only in the core microbiota of SUP+ piglets.

To evaluate if the taxonomies correlated with the VFAs found in the feces, we performed multivariate association analyses on the taxonomies found in all the samples, independently by age and treatment. We found that five OTUs correlated with acetate, two OTUs with iso-butyrate, and three different OTUs correlated with propionate, butyrate, and valerate, respectively (*p* < 0.001; Table 4).

## 4. Discussion

### 4.1. Effect of Supplementation on Growth Performance and Health

In the present experiment, the supplementation given only at birth resulted in an improved post-weaning ADG, leading to a 1.2 kg greater body weight two weeks post-weaning. This effect may be related to the combination of the two strains of probiotics. Previous studies reported a positive effect of a repeated probiotic supplementation either with *E. faecium* or *S. cerevisiae* on piglet growth [21,22]. The administration of 6 × 10^8^ CFU/mL of *E. faecium* on the first, third, and fifth days after birth improved pre- and post- weaning growth [21]. Similarly, a daily intake of 5 × 10^9^ or 2.5 × 10^10^ CFU of *S. cerevisiae* for the whole suckling period improved the ADG of suckling piglets by 30 g/d and weaning weight by 1 kg [22]. In the present study, besides being applied at greater doses, the two probiotic strains were effective on piglet growth when supplied only at birth. This beneficial effect may come from improved intestinal health in SUP+ piglets, as these piglets developed less diarrhea in the first two weeks post-weaning. Both probiotics have been shown to be effective against post-weaning diarrhea [21,23]. Bajagai et al. reported that probiotics stimulate immunity by increasing immunoglobulin production [24]. Moreover, inulin may also affect health. The aforementioned prebiotic stimulates immune function by potentiating an inflammatory response [25]. Owing to their involvement in immune processes, vitamins and selenium in the supplement may also have contributed to the reduction of diarrhea [16,26,27]. In their recent review, Matte and Audet demonstrated that, within the first week of life, the requirements of selenium and vitamins A and D cannot be covered with sow milk only [28]. As lactation diets contain great amounts of vitamin E, it is not considered to be a limiting factor in colostrum and in milk [29]. Nevertheless, piglets unable to suckle enough colostrum may have poorer immune system development. Therefore, supplementation with vitamins and selenium at birth might also help to enhance piglet immunity.

### 4.2. Effect of Supplementation on Fecal Microbiota and Fermentation

Milk consumption is a critical step for gastro-intestinal tract maturation. In addition, oligosaccharides, amino acids, and other bioactive compounds that are normally present in milk can strongly affect the gut microbiota of suckling piglets, since the microbiota is less diverse in the suckling period as well as in the weaning period [30]. Due to nutritional, environmental, and social upheaval, weaning is a stressful period in a piglet’s life. The separation from the mother and littermates, together with a new environment and the introduction of solid feed of plant origins, can lead to sub-optimal growth and, simultaneously, to profound physiological, immunological, and microbiological changes. Therefore, the intestinal structure and functionality, as well as the gut microbiota, of the piglet is strongly affected [31]. During the first week post-weaning, the microbiota becomes highly unstable with a strong decrease in biodiversity, which is normally restored after two or three weeks [31,32,33]. Accordingly, our results showed a lower abundance and biodiversity of microbial composition during the suckling period compared to two weeks post-weaning, in both SUP+ and SUP− piglets. 

The use of probiotics, prebiotics, or polyphenols [34,35,36] or a combination of all three is a nutritional strategy used to prevent pathogenic infections in monogastric organisms by modulating the gut microbiota composition [37]. In our study, the single-dose supplementation of *E. faecium* and *S. cerevisiae* strains, together with prebiotics, did not influence either the abundance or biodiversity of the microbial community, compared to the SUP− group. However, the supplementation affected some less-abundant taxa. The LEfSe was performed to discover distinctive taxa at all taxonomic levels between SUP+ and SUP− piglets. This analysis shows that, in 16-day-old piglets, after synbiotic supplementation, potential biomarkers of gut health, such as the presence of the Saccharibacteria and Cyanobacteria phyla, together with the Lentisphaeria class, might be considered. Due to their difficulty to be grown in conventional cultivation, Saccharibacteria physiology and their role in health and diseases is still poorly described. However, it is known that their main energy sources are sugar compounds, and that lactate is the main carbohydrate source of these bacteria [38]. Even if they are rare and ultrasmall, Saccharibacteria can potentially affect the ecology of the microbial community and the physiology of its host [38]. Accordingly, associations between Saccharibacteria and a healthy gut are reported in the literature [39]. It has been suggested that Saccharibacteria may be involved in carbohydrate utilization, which may have been beneficial for the SUP+ piglets [40]. Together with bacteria from the Saccharibacteria phylum, Cyanobacteria bacteria and bacteria associated with this phylum (such as those of the Gastranaerophilales order) decreased in inflammatory animal models, such as diarrhea in irritable bowel syndrome mice [41] and diet-induced obese mice [42]. Thus, an increased relative abundance of those bacteria in the SUP+ piglets could be considered protective for the gut health of the piglets. In our study, bacteria from the *Victivillaceae* family and Victivillales order, belonging to the Lentisphaeria class, were more abundant in the SUP+ piglets. The study results demonstrated that this class of bacteria may be involved in the nutrient metabolism of the host, especially in the metabolism of carbohydrates, peptides, and amino acids, and affecting protein utilization and providing beneficial metabolites [43]; thus, their presence is considered beneficial for the host [43]. 

Surprisingly, in older SUP+ piglets, the abundance of bacteria from the Verrucomicrobiae class and *Akkermansia* genus was reduced compared to SUP− piglets. Previous reports have shown a reduced proportion of these bacteria in diarrheal subjects, which is considered a characteristic dysbiosis associated with diarrhea [44]. In addition, prebiotics normally increase the abundance of bacteria belonging to the *Akkermansia* genus, such as *Akkermansia muciniphila* [42]. *A*. *muciniphila* is able to improve mucus production using goblet cells and improve barrier function [42]. However, in the present study, the presence of those taxa was not related to the health status of the piglets. 

It has been reported that, compared to healthy subjects, a smaller core microbiota is usually observed in unhealthy individuals, suggesting a loss of some health-associated core bacteria [45]. In the present study, SUP+ piglets had a core microbiota composed of 15 different OTUs, while only nine OTUs composed the core microbiota of the SUP− group during the suckling period. Compared to the weaning period, the sow milk–based suckling stage favored the growth of bacteria belonging to the Ruminococcus phylum as early gut colonizers, independent of the supplementation. However, important differences have been found in this period between SUP+ and SUP− piglets, specifically, the presence of bacteria from the *Lactobacillus* genus. This genus has been previously identified as a growth-related taxa during the nursery phase and promoters of animal growth [30]. In addition, these bacteria have been reported to decrease epithelial permeability and improve barrier function [30]. As a consequence, the presence of bacteria from the *Lactobacillus* genus and a more diverse core microbiota observed after supplementation on Day 16 of age, could have led to a reduced occurrence of post-weaning diarrhea in this study group. The introduction of solid nutrients strongly influenced core microbiota composition. Two weeks post-weaning, the core microbiota of both groups of piglets was dominated by members of the *Prevotella* genus, undetectable during the suckling phase. This is in line with the literature, since members of *Prevotella* are associated with plant-based diets and fiber digestion [46]. Interestingly, in this period, the core microbiota of both SUP+ and SUP− piglets were very similar and were composed of 9 OTUs. The decreased OTU number in the core microbiota of SUP+ piglets over time suggests that the strongest effects of the supplementation are probably exerted during the suckling period, which is probably the most important for proper gut development. 

The supplementation did not affect the production of VFAs during the experiment. The higher abundance of total VFAs in post-weaning piglets compared to milk-fed piglets is in line with gut microbiota results, where a greater microbial abundance and biodiversity leads to an increased production of those fermentation products. Interestingly, members of the core microbiota seem to correlate with the production of specific VFAs. Acetate, for example, was more abundant in the post-weaning period, and it was negatively correlated with members of the Clostridiales order, the *Bacteroides* genus, and the *Ruminococcaceae* family, which are part of the core microbiota in the suckling period. In contrast, the abundance of members from the *Alloprevotella* genus, including members of the *Prevotelaceae* family, that dominate the core microbiota in post-weaning piglets, was positively correlated with the production of the same VFAs. Similarly, iso-butyrate was more abundant in the suckling period and positively correlated with the abundance of members of the *Alistipes* genus, which were found in the core microbiota of SUP+ piglets in the same period.

## 5. Conclusions

The present study shows that a supplementation of synbiotics, vitamins, selenium, and immunoglobulins given at birth improved the growth performance of piglets after weaning, and decreased the occurrence of diarrhea, in particular in the first week post-weaning. The enhanced growth performance, together with the reduction of diarrhea, seems to be associated with changes in the fecal microbiota during the suckling period, rather than at two weeks post-weaning. Indeed, at 16 days of age, the supplementation did not affect microbial abundance and diversity of the main taxa, but affected some less-abundant taxa, such as bacteria from the Saccharibacteria and Cyanobacteria phyla, and the Lentisphaeria class. In addition, the greater core microbiota of SUP+ piglets, particularly the presence of members of the *Lactobacillus* genus during the suckling period, may have contributed to better health. This study stresses the importance of early-life interventions to improve piglet development and health in the post-weaning period.

## Figures and Tables

**Table 1:** Growth performance and occurrence of diarrhea in piglets from the unsupplemented (SUP−) and supplemented (SUP+) groups. The supplement, given at birth, consisted of a mixture of probiotics, prebiotics, immunoglobulins, vitamins, and selenium.

	SUP−	SUP+	SEM	*p*-Value
Average daily gain (g/d)				
d 0–25	226	225	15.3	0.98
d 25–32	−96	−22	23.6	0.04
d 32–39	180	249	37.6	0.20
d 0–39	159	185	8.8	0.05
Occurrence of diarrhea (%)				
d 18–25	3.3	6.3	1.87	0.36
d 25–32	26.4	12.5	4.62	0.03
d 32–39	25.0	15.6	4.25	0.10
Total	18.5	11.7	2.30	0.02
Days of diarrhea	4.4	3.0	0.84	0.22

**Table 2:** Volatile fatty acid content in the feces of piglets from the unsupplemented (SUP−) or supplemented (SUP+) groups on Day 16 of age and two weeks post-weaning (39 ± 1 d). The supplement, given at birth, consisted of a mixture of probiotics, prebiotics, immunoglobulins, vitamins, and selenium.

Day	16		39 ± 1			*p*-Value		
Group	SUP−	SUP+	SUP−	SUP+	SEM	SUP ^1^	Day	SUP × Day
Total (mol/g)	40.3	56.2	86.1	93.0	6.86	0.14	< 0.001	0.40
Proportion (%)								
acetate	52.6	52.7	63.0	62.9	1.91	0.98	< 0.001	0.96
propionate	17.2	17.9	22.3	20.7	0.91	0.63	< 0.001	0.15
butyrate	15.1	15.3	10.6	12.1	1.36	0.47	< 0.01	0.66
isobutyrate	4.71	4.3	1.1	1.2	0.26	0.52	< 0.001	0.26
valerate	3.4	3.1	1.8	2.0	0.30	0.96	< 0.001	0.63
isovalerate	7.0	6.6	1.1	1.1	0.46	0.65	< 0.001	0.47

^1^ SUP: effect of the supplementation.

**Table 3:** Discriminative bacteria associated with supplementation in probiotics, prebiotics, immunoglobulins, vitamins, and selenium on 16 days of age and two weeks post-weaning.

Day	Taxonomic Rank	OTU ^1^	Taxa	Log2 Fold CHANGE	*p*-Value
16	genus	154	*Candidatus Saccharimonas*	6.33	0.023
	order	508	Gastranaerophilales	2.37	0.011
	family	196	*Victivillaceae*	4.23	0.004
	genus	830	*Butyricimonas*	−2.24	0.032
42	genus	83	*Intestinimonas*	−3.14	0.024
	genus	122	*Akkermansia*	−9.48	0.023
	genus	152	*Anaerotruncus*	NA	0.008

^1^ OTU: operational taxonomic unit. NA: The Log2 Fold change cannot be estimated as the bacteria of the related genus were found only in one group.

**Table 4:** Multivariate linear regression analysis of gut microbiota relative abundancies and volatile fatty acid concentrations found in feces ^1^.

OTU	Taxa	VFA	N	N Not 0	CoE	*p*-Value	Q-Value
994	*Ruminiclostridium_9*	Total	56	37	−7.5 × 10^−4^	2.3 × 10^−5^	0.017
994	*Ruminiclostridium_9*	Acetate	56	41	−9.6 × 10^−4^	2.2 × 10^−5^	0.002
292	*Bacteroides*	Acetate	56	18	−1.2 × 10^−4^	1.3 × 10^−4^	0.011
60	*Ruminococcaceae_UCG−002*	Acetate	56	56	−1.4 × 10^−3^	3.6 × 10^−4^	0.023
558	*Alloprevotella*	Acetate	56	37	9.4 × 10^−4^	6.5 × 10^−4^	0.037
224	*Alistipes*	Iso-butyrate	56	42	6.7 × 10^−3^	4.2 × 10^−5^	0.004
94	*Thalassospira*	Iso-butyrate	56	26	6.9 × 10^−3^	3.1 × 10^−4^	0.021
994	*Ruminiclostridium_9*	Butyrate	56	41	−1.2 × 10^−3^	5.3 × 10^−5^	0.005
516	*Helicobacter*	Propionate	56	42	8.7 × 10^−4^	5.1 × 10^−4^	0.029
623	*Prevotella_2*	Valerate	56	27	6.9 × 10^−3^	2.5 × 10^−4^	0.018
94	*Thalassospira*	Iso-valerate	56	26	6.9 × 10^−3^	4.9 × 10^−4^	0.029

^1^ Taxa are presented at the genus level. OTU: operational taxonomic unit; VFA: volatile fatty acid; N: number of samples analyzed; N not 0: number of samples in which the abundance of the related taxa is higher than zero; CoE: correlation coefficient. Data were obtained through multivariate analysis by linear models (MaAsLin).

## Data Availability

The data presented in this study are available on request from the corresponding author.

## Supplementary Materials

The following are available online at https://www.mdpi.com/2076-2615/11/1/84/s1, Figure S1: Alpha diversity indexes of the gut microbiota population in supplemented (SUP+) and unsupplemented (SUP−) piglets on Day 16 of age (d16) and at two weeks post-weaning (d39). Figure S2: Weighted and unweighted UniFrac beta-diversity analysis of gut microbiota in supplemented (SUP+) and unsupplemented (SUP−) piglets on Day 16 of age (d16) and at two weeks post-weaning (d39). Figure S3: Linear discriminant analysis coupled with effect size measurements (LEfSe) of piglets on Day 16 of age (d16) and at two weeks post-weaning (d39).
