# Supplementary figures and images for: A Single Dose of Synbiotics and Vitamins at Birth Affects Piglet Microbiota before Weaning and Modifies Post-Weaning Performance
Source: Animals (Basel). 2021 Jan 5;11(1):84. doi: 10.3390/ani11010084 (PMC7824832; doi:10.3390/ani11010084)

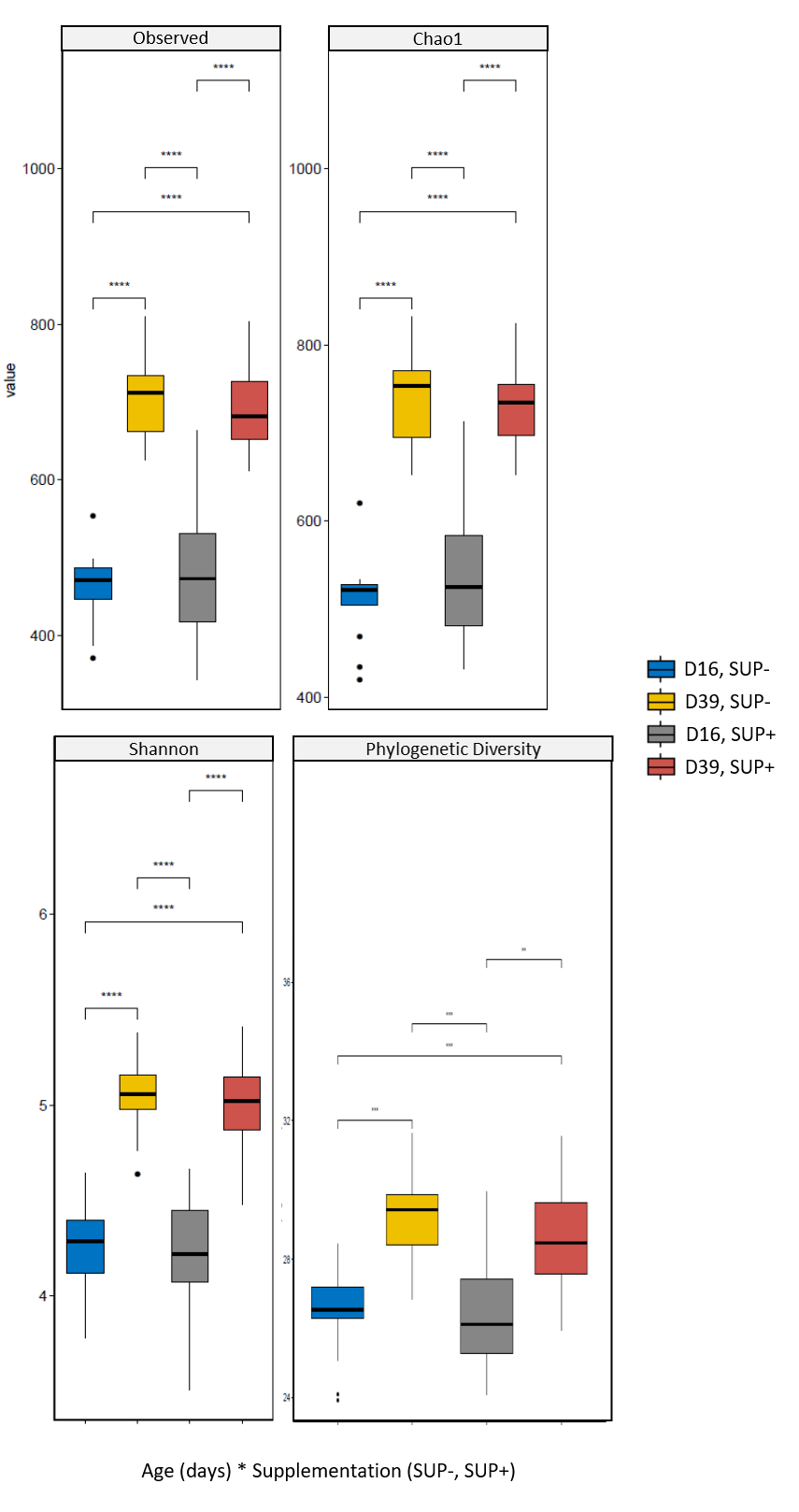

Supplement: Supplementary file 1 [file animals-11-00084-s001.zip › Supplementary material_Girard et al/Supplementary_1.tif]

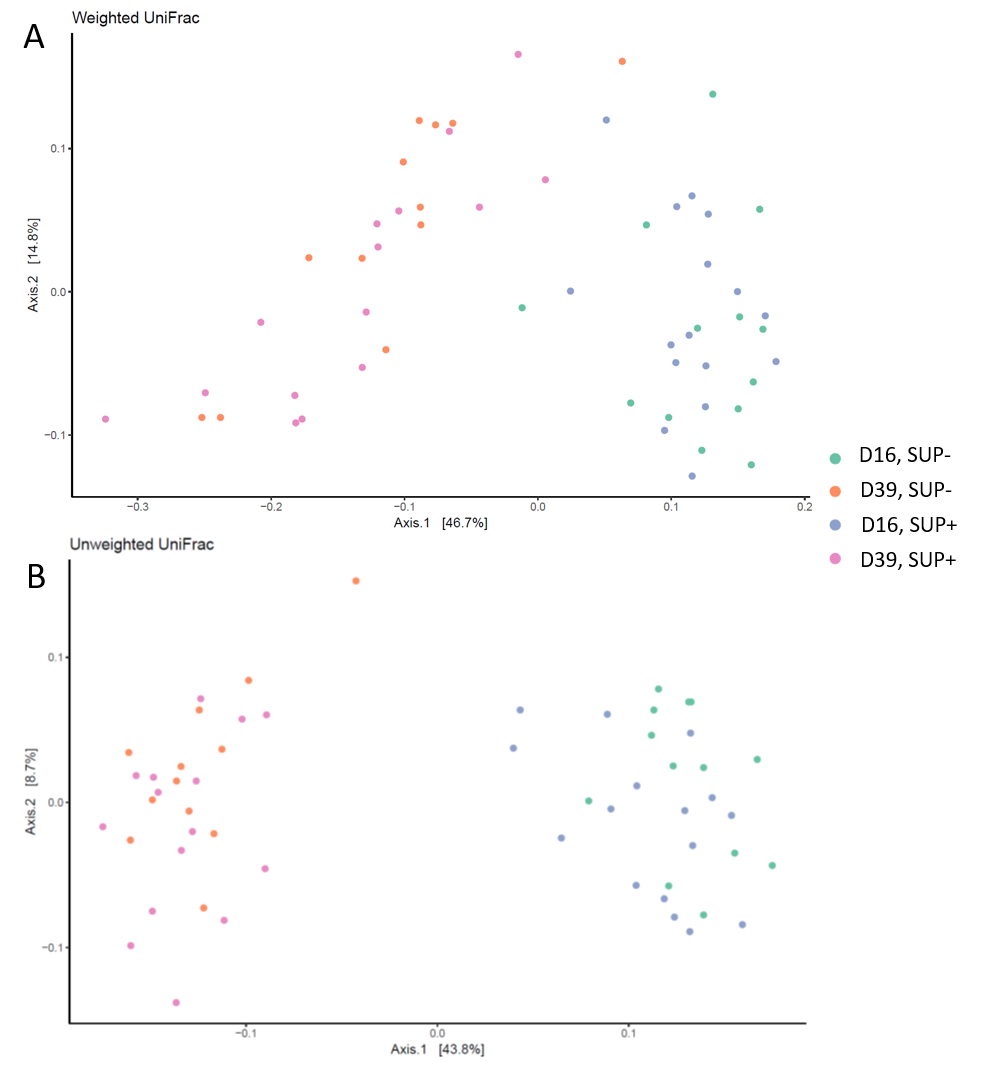

Supplement: Supplementary file 1 [file animals-11-00084-s001.zip › Supplementary material_Girard et al/Supplementary_2.tif]

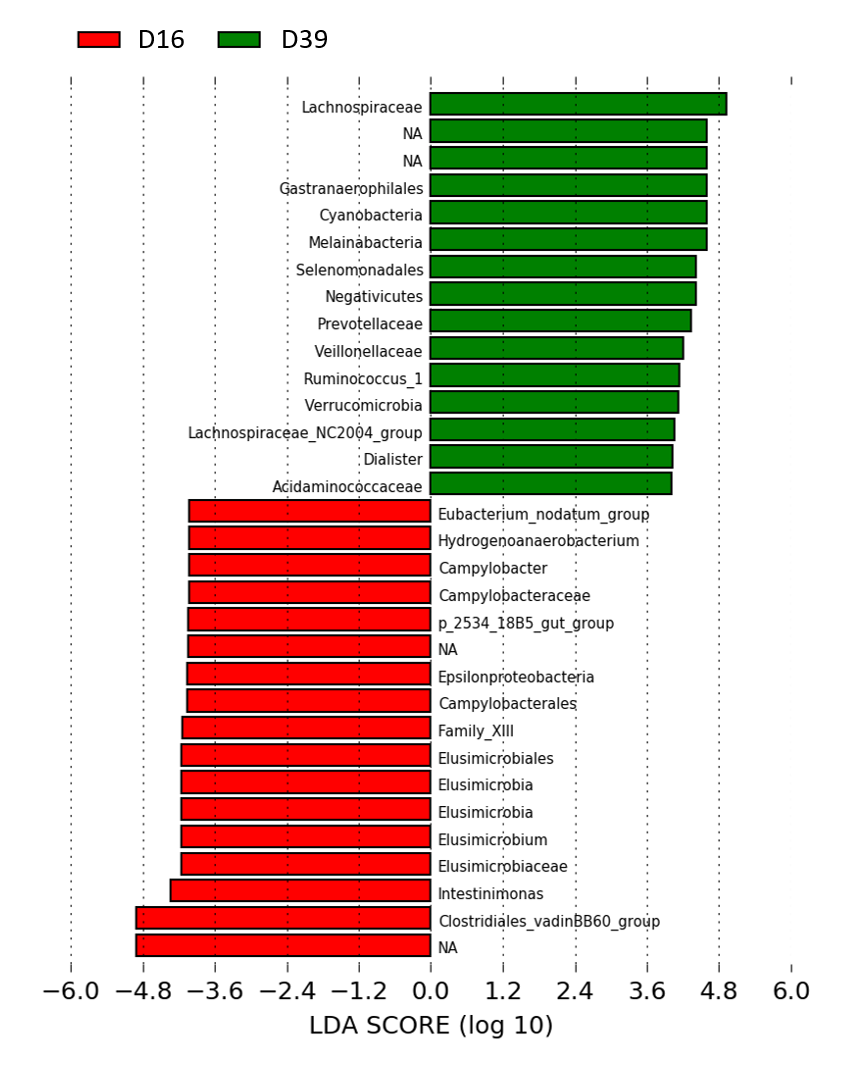

Supplement: Supplementary file 1 [file animals-11-00084-s001.zip › Supplementary material_Girard et al/Supplementary_3.tif]
